# Accelerating the manufacturing of HIV Env protein vaccines for early phase clinical evaluation

**DOI:** 10.1038/s41541-025-01169-y

**Published:** 2025-08-20

**Authors:** Michael N. Pensiero

**Affiliations:** National Institute of Allergy and Infectious Diseases, Rockville, MD USA

**Keywords:** Biotechnology, Recombinant vaccine

## Abstract

This commentary discusses the benefits in terms of time savings and reduced costs of goods in using transient electroporation and immuno-affinity purification as an alternative to the traditional manufacturing approaches for the CGMP manufacturing of HIV Envelope protein immunogens for use in investigational clinical studies. The companion manuscript by Bale et al. describes in detail this approach for CGMP manufacture and analytical evaluation of a near-native HIV envelope timer.

HIV-1 poses a significant health risk; therefore, the development of a vaccine targeting the surface glycoprotein (Env) is a top priority to reduce the disease burden^1,2^. Rapid clinical evaluation of Env candidates (gp120 monomers, gp120 outer domain/cores, gp140 soluble trimers or nanoparticles containing HIV trimers) would be facilitated by the development of a generalizable manufacturing process designed to accelerate the timescale and reduce cost for upstream (bioreactor) and downstream (cell purification steps) CGMP (current Good Manufacturing Practices) manufacturing processes for HIV-1 Env immunogen-based vaccines. The Vaccine Research Program (VRP)/Division of AIDS (DAIDS)/NIAID at NIH initiated a contract with Advanced Science Biolaboratories (ABL) (funded under NIAID contracts: HHSN272201100021I and HHSN272201700010I) towards developing a feasible, more efficient, and cost-effective alternative for CGMP manufacturing over the more traditional development of a cloned Master Cell bank (MCB) followed by multi-step downstream purification process via ion exchange chromatography. Towards that objective, VRP explored whether the use of transient expression via electroporation for upstream manufacturing and immuno-affinity purification for downstream manufacturing processes was feasible for CGMP manufacturing of three HIV Env proteins as vaccine candidates. In addition to ABL’s role as the prime contractor (upstream manufacturing via transient transfection), the other major funded partner organizations involved in this study included the International AIDS Vaccine Initiative (IAVI) (immuno-affinity preclinical evaluation funded under contract HHSN272201600011I), MaxCyte (electroporation), KBI (downstream process development), and the Duke Human Vaccine Institute (downstream manufacturing). This new approach shifts design and production parameters from classical stable cell line or cell pool development to gram quantity immunogen expression by electroporation-based transient transfection at a 50–80 L bioreactor scale. The overall production timelines to starting Upstream CGMP would also be reduced from a minimum of twelve months (stable cell line) or 6 months (stable cell pool)^3^ to about four months (transient electroporation) when all the independent processes are performed in parallel (Fig. 1). Until recently, transient upstream manufacturing was not considered as a viable option for generation of HIV Env proteins. However, HIV Env protein (eOD-GT8) manufacturing using chemical transfection has been recently described using HEK293 cells at a 200 L scale with yields of 354 mg of final drug product^4^. The transient approach using electroporation (versus chemical transfection) described in the companion manuscript and below report a scale of ~80 L with yields of drug product on the order of grams; thus, in addition to the potential time savings, this platform also lowers the overall cost of goods (COG). The initial success of the electroporation approach was demonstrated by the upstream CGMP production of an HIV-1 Env CH505 gp120 monomer with attributes biochemically and antigenically identical to the same glycoprotein monomer expressed from stable pools or a stable master banked CHO (Chinese hamster ovary)-based cell line used in a clinical study (HVTN 115, NCT03220724). The transiently produced CH505TF (Transmitted Founder) gp120 monomer was compared to the equivalent stably produced version of the CH505TF gp120 monomer protein in a human clinical trial (HVTN 123, NCT03856996). DAIDS then supported and sponsored the production of a complexly designed, near-native HIV-1 Env gp140 trimer derived from an Indian clade C HIV-1 isolate designated as stabilized 16055 DG4 NFL (developed in the Wyatt laboratory)^5^. The companion manuscript (Bale et al.) describes the accelerated CGMP production of the structure-based stabilized 16055 DG4 NFL. The paper describes transient expression from electroporation of CHO-S cells and downstream affinity chromatography purification using a broadly neutralizing HIV antibody, PGT145, conjugated to a Toyopearl AF-Tresyl-650M resin for manufacture of the 16055 DG4 NFL at a reduced time frame. The paper also describes evaluation of the pre-GMP process development or engineering run material by differential scanning calorimetry, antigenic profiling, and electron microscopy. A major strength of the study lies in its detailed characterization of the 16055 DG4 NFL trimers manufactured by the CGMP process. The authors employed robust biophysical and structural validation methods, including cryo-electron microscopy, to confirm the trimer’s conformational integrity. The final products were demonstrated to be stable and displayed favorable biophysical characteristics of a well-folded soluble trimer. This HIV-1 stabilized vaccine candidate trimer is currently being evaluated in a phase I human clinical trial for safety and immunogenicity (HVTN 313, NCT06332339). To further test the utility of electroporation/immuno-affinity purification in a CGMP setting, DAIDS undertook the manufacturing of multilayered single-component self-assembling protein nanoparticle displaying 20 stabilized BG505 Env trimers (BG505-UFO-E2P SApNP, original sequence provided by Jiang Zhu/ at Scripps Research/Uvax Bio). Building on the success from the previous monomer and soluble trimer CGMP campaigns, the electroporation upstream process was further optimized, and a CGMP campaign for BG505-UFO-E2P SApNP at a 80 L upstream bioreactor scale was completed which, resulted in up to 8.7 g upstream harvest and ~5 g of purified of final DP (drug product). The data to date demonstrate that transient transfection via electroporation followed by immune-affinity DS (drug substance) purification allow for the efficient manufacturing of gp120 HIV Envs, soluble well-folded gp140 trimers, and gp140-self-assembling NP at a manufacturing scale between 50-80L with yields suitable for testing in Phase I clinical studies; moreover as presented in the Bale et al. manuscript, the products appear homogenous and of sufficient quality, addressing an initial concern that HIV Env proteins derived from a transient approach would result in a heterogenous product or a product that was degraded due to the electroporation process. Two of the three products have already entered clinical studies. The upstream process reported in the companion paper has precluded the need for development of an MCB, which saved both cloning and down-selection time and considerable costs (>$500 K) for manufacturing and release of the final MCB. The transient transfection platform process described above is now based on the maintenance of a single cell substrate, CHO-S, for the manufacture of multiple HIV Env immunogens, versus having to generate a separate MCB for each HIV Env immunogen. The electroporation approach could be used for manufacturing of multiple HIV immunogens in early iterative Phase clinical studies. It is not clear to the extent the transient electroporation would provide consistent results at larger scales (>80 L) or provide sufficient product consistency suitable for later Phase clinical studies. It is envisioned that the approach described in the accompanying publication could be expanded to other antigens of interest and could significantly accelerate CGMP production process timelines.

**Fig. 1:**
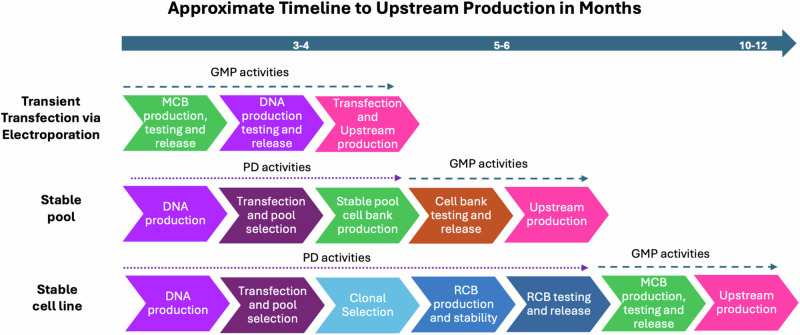
Comparison of approximate timelines to Upstream CGMP Production of HIV Env proteins for Electroporation vs Stable Pool vs Stable Cell lines, delineating the time for Process Development (PD) activities in a non-GMP environment and CGMP activities.

## Data Availability

No datasets were generated or analysed during the current study.
